# Novel Human Rotavirus Genotype G5P[7] from Child with Diarrhea, Cameroon 

**DOI:** 10.3201/eid1501.080899

**Published:** 2009-01

**Authors:** Mathew D. Esona, Annelise Geyer, Krisztian Banyai, Nicola Page, Maryam Aminu, George E. Armah, Jennifer Hull, Duncan A. Steele, Roger I. Glass, Jon R. Gentsch

**Affiliations:** Centers for Disease Control and Prevention, Atlanta, Georgia, USA (M.D. Esona, J. Hull, J. Gentsch); University of Limpopo, Pretoria, South Africa (A. Geyer); Association of Public Health Laboratories, Washington, DC, USA (K. Banyai); National Institute for Communicable Diseases, Johannesburg, South Africa (N. Page); Ahmadu Bello University, Zaria, Nigeria (M. Aminu); Noguchi Memorial Research Institute, Accra, Ghana (G.E. Armah); Program for Appropriate Technology in Health, Seattle, Washington, USA (D.A. Steele); National Institutes of Health, Bethesda, Maryland, USA (R.I. Glass); 1 Member of the African Rotavirus Network.

**Keywords:** Rotavirus, surveillance, reassortment, Cameroon, dispatch

## Abstract

We report characterization of a genotype G5P[7] human rotavirus (HRV) from a child in Cameroon who had diarrhea. Sequencing of all 11 gene segments showed similarities to >5 genes each from porcine and human rotaviruses. This G5P[7] strain exemplifies the importance of heterologous animal rotaviruses in generating HRV genetic diversity through reassortment.

Group A rotaviruses are a major cause of severe diarrheal disease in infants, young children, and a variety of animals. In humans, rotavirus gastroenteritis results in deaths and hospitalizations; most deaths have occurred in developing countries (*1*).

Rotavirus surveillance and strain characterization, in support of rotavirus vaccine development programs, have detected many new human rotavirus (HRV) genotype specificities and highlighted the importance of mechanisms such as reassortment and zoonotic transmission in the evolution of rotaviruses (*2*). However, more comprehensive analyses of gene fragments (*3*) or entire genes (*4*) are needed to clarify the origin of rotavirus gene segments for common and uncommon strains. To elucidate the possible origin of the novel G5P[7] HRV strain from the African Rotavirus Surveillance Network (ARN), we determined its genomic composition and compared its gene sequences with rotavirus sequences in GenBank.

## The Study

During ARN surveillance conducted from 1998 through 2004, a total of 215 rotavirus-positive stool samples could not be typed by standard reverse transcription–PCR genotyping methods. Among untypeable samples, we identified a G5P[7] strain (designated 6784/2000/ARN), which represented a rare G genotype and a new P genotype specificity in humans. This strain was isolated from a stool specimen from a child with gastroenteritis in Kumba, Cameroon. Because G5 and P[7] genotype specificities are common in pigs, we studied the entire genomic composition of this strain to determine if it was an example of a strain that arose through direct interspecies transmission from a particular animal host, or by reassortment with heterologous rotavirus strains.

Gene fragments of the 11 gene segments of strain 6784/2000/ARN were amplified by using consensus primers for structural protein 4 (VP4), VP6, and VP7 (*5**–**8*) and newly designed consensus primers for VP1, VP2, VP3, nonstructural protein 1 (NSP1), NSP2, NSP3, NSP4, and NSP5 (Table 1). The fragments were sequenced by using the BigDye Terminator Cycle Sequencing Kit (Applied Biosystems, Foster City, CA, USA). Dye-labeled products were sequenced in an ABI 3130 sequencer (Applied Biosystems). Similarity and phylogenetic relationships were inferred by using aligned nucleotide and deduced amino acid sequences by the neighbor-joining method and p-distance algorithm of MEGA4 software (*9*).

**Table 1:** Primers used for amplification and sequencing of rotavirus genes

Primer*	Sequence (5′ → 3′)†	Gene‡	Nucleotide position, strand	Amplicon size, bp	Strain	Reference
MDEVP1F	AAT CAC AAT CTG CAG TTC AAA	VP1	68–­89, +	337	Ku	This article
MDEVP1R	AAT GAA TCA GTG TAT TCT TCG	VP1	405–384, –		Ku	This article
MDEVP2F	CTG ACA AAG TGC TAT CAC A	VP2	156–175, +	300	Ku	This article
MDEVP2R	AGG TAA TTG TCT TGG TTC	VP2	456–438, –		Ku	This article
MDEVP3F	TTG CTA GAT TGT CAA ATC GTG	VP3	597–618, +	327	Ku	This article
MDEVP3R	AAT AAG ATG GAG CTG AAC C	VP3	924–905, –		Ku	This article
MDENSP1F	GAG ACC RTC AAC TCC TAC YAA	NSP1	120–141, +	344	Wa	This article
MDENSP1R	ATT GTA AYG TTA TTG GCA T	NSP1	464–445, –		Wa	This article
MDENSP2F	GCT TGC TTT TGT TAT CCT	NSP2	58–76, +	327	Ku	This article
MDENSP2R	ATT TTC CAA ATG TCT AAC AG	NSP2	385–365, –		Ku	This article
MDENSP3F	GCC ACT TCA ACA TTA GAA	NSP3	101–119, +	303	Ku	This article
MDENSP3R	TAC ACT AAA ACA AGC ATT AAG	NSP3	404–383, –		Ku	This article
MDENSP5F	AGC GCT ACA GTG ATG TCT CT	NSP5	10–29, +	337	Ku	This article
MDENSP5R	CCA TTT GAT CGC ACC CA	NSP5	347–330, –		Ku	This article
JRG30	GGC TTT TAA AAG TTC TGT T	NSP4	1–19, +	737	Wa	This article
JRG31	ACC ATT CCT TCC ATT AAC	NSP4	738–721, –		Wa	This article
Con3	TGG CTT CGC TCA TTT ATA GAC A	VP4	11–32, +	876	Ku	(*5*)
Con2	ATT TCG GAC CAT TTA TAA CC	VP4	887–868, –		Ku	(*5*)
9con1-L	TA GCT CCT TTT AAT GTA TGG TAT	VP7	37–59, +	896	Wa	Modified from (*6*)
VP7-Rdeg	GAC GGV GCR ACT ACA TGG T	VP7	933–914, –		Wa	Modified from (*7*)
VP6-F	GAC GGV GCR ACT ACA TGG T	VP6	747–766, +	379		(*8*)
VP6-R	GTC CAA TTC ATN CCT GGT G	VP6	1126–1106, –			(*8*)

*F, forward; R, reverse. †R, A or G; Y, C or T; V, A, C, or G; N, A, C, G, or T. ‡VP, structural protein; NSP, nonstructural protein.

Similarity matrices and phylogenetic trees based on nucleotide and amino acid sequences were constructed and compared with cognate gene sequences of human and animal rotaviruses. Except for the 2 gene segments, which encode neutralizing antigens VP7 and VP4, respectively, and are commonly encountered in porcine rotaviruses, the remaining 9 gene segments of 6784/2000/ARN were grouped in a common phylogenetic clade in which reference human strains of the Wa genogroup and related porcine rotaviruses also clustered (Appendix Figure). However, VP1, NSP3 (likely), and NSP5 genes were more closely related to cognate gene sequences of porcine strains (Gottfried, PRICE, CMP034, and OSU) than to HRVs and shared an nt identity of 92%–99%. VP2, VP3, VP6, NSP1, NSP2, and NSP4 genes showed a stronger genetic relationship with human strains of the Wa genogroup (90%–99% nt identities) than with known porcine rotaviruses (Table 2).

**Table 2:** Nucleotide/amino acid identities of rotavirus 6784/2000/ARN gene segments with cognate gene sequences of 36 known human and animal rotavirus sequences from GenBank*

Strains†	Nucleotide/amino acid identity, %										
	VP1	VP2	VP3	VP4	VP6	VP7	NSP1	NSP2	NSP3	NSP4	NSP5
Ku/G1P[8]/Hu	88/94	**96**/96	90/94	61/55	90/98	74/79	85/88	**91**/94	94/94	89/93	94/96
DRC88/G8P[8]/Hu	79/85	76/77	69/73	–	76/93	74/81	74/75	85/93	84/90	79/83	90/93
OSU/G5P[7]/Po	–	–	86/90	**90**/89	80/90	86/94	86/89	–	89/95	88/96	**98**/100
RMC321/G9P[19]/Hu	82/93	84/94	–	–	80/91	–	81/87	88/91	89/93	88/95	97/98
Tb-chen/G2P[4]/Hu	79/83	77/79	69/72	62/56	77/91	–	77/76	88/92	85/90	81/83	89/92
AU-1/G3P[9]/Hu	78/84	77/84	74/76	59/56	76/91	78/85	69/72	81/89	82/93	78/82	93/97
ST-3/G4P[6]/Hu	–	–	91/94	–	–	–	94/94	–	97/97	88/91	–
69M/G8P[10]/Hu	–	–	71/75	–	–	–	77/76	–	82/88	–	94/95
T152/G12P[9]/Hu	–	–	–	60/57	76/91	75/82	70/71	–	–	–	–
R14a/G9P[8]/Hu	–	–	–	–	–	–	**97**/97	–	–	–	–
Wa/G1P[8]/Hu	85/94	93/93	**95**/96	62/55	88/98	74/78	85/88	88/91	97/97	**90**/95	95/95
DS-1/G2P[4]/Hu	79/84	–	70/75	62/56	79/92	72/74	76/76	88/94	83/89	81/84	–
30/96/G3P[14]/Lp	79/85	79/85	73/82	61/56	–	–	–	86/95	83/90	79/83	91/96
PRICE/Po	–	–	–	–	–	–	–	–	**98**/98	–	–
RRV/G3P[3]/Si	–	–	–	69/72	–	–	–	–	82/92	79/82	–
PO-13/G18P[17]/Av	67/61	63/56	62/57	39/20	70/77	65/58	–	56/46	60/58	–	63/52
KJ75/G5P[5]/Bo	–	–	–	–	–	86/93	–	89/94	–	–	–
US1205/G9P[6]/Hu	–	–	–	61/57	76/91	77/84	–	–	–	80/83	–
EW/G16P[16]/Mu	–	–	–	62/60	–	–	–	–	–	62/62	–
KUN/G2P[4]/Hu	–	–	–	–	–	–	–	–	–	81/83	90/93
CU-1/G3P[3]/Ca	–	–	–	–	–	–	–	–	–	78/84	–
FRV-1/G3P[9]/Fe	–	–	–	–	–	–	–	–	–	79/82	–
EHP/G16P[20]/Mu	–	–	–	65/66	–	–	–	–	–	62/61	–
SA-11/G3P[1]/Si	77/85	79/84	–	71/72	–	–	–	–	–	–	94/96
CMP034/G2P[27]/Po	–	–	–	–	–	–	–	–	–	–	**99/100**
YM/G11P[7]/Po	88/94	–	–	85/88	81/90	82/90	–	–	–	–	–
Gottfried/G4P[6]/Po	**92**/96	–	–	61/57	88/98	–	–	–	–	–	–
JL94/G5P[7]/Po	–	–	–	**90**/89	–	–	–	–	–	–	–
rj6906/03/Hu	–	–	–	–	**98**/99	–	–	–	–	–	–
MRC3105/G5P[8]/Hu‡	–	–	–	–	–	**100**/100	–	–	–	–	–
CC117/G5/Po	–	–	–	–	–	91/97	–	–	–	–	–
C134/G5/Po	–	–	–	–	–	90/97	–	–	–	–	–
LL4260/G5P[6]/Hu	–	–	–	–	–	90/94	–	–	–	–	–
KH210/G5P[6]/Hu	–	–	–	–	–	89/93	–	–	–	–	–
IAL-28/G5P[8]/Hu	–	–	–	–	–	85/92	–	–	–	–	–
H-1/G5P[7]/Eq	–	–	–	–	81/90	86/94	–	–	–	85/93	–

*ARN, African Rotavirus Surveillance Network; VP, structural protein; NSP, nonstructural protein; –, not included or not sequenced. High and moderate nucleotide/amino acid percentage identities are in **boldface**. †Species of origin. Hu, human; Po, porcine; Lp, lapine; Si, simian; Av, avian; Bo, bovine; Ca, canine; Fe, feline; Mu, murine; Eq, equine. ‡VP7 gene of MRC3105 was derived from a porcine rotavirus.

Sequence analysis of the VP7 gene demonstrated that 6784/2000/ARN had 85%–91% nt and 92%–100% aa identities with representative G5 rotaviruses from humans and animals, respectively. Although the VP7 gene was highly divergent from other human G5 isolates detected in South America and Asia, it was identical to a human serotype G5 rotavirus isolated in Cameroon (*10*) and clustered with 2 porcine strains from Argentina (Appendix Figure). Genetic analysis of the VP8* portion of the VP4 gene of strain 6784/2000/ARN had higher similarity (90% nt and 89% aa) with porcine genotype P[7] strains, e.g., OSU and JL94, than with strains of other genotypes (39%–85% nt and 55%–72% aa). This finding suggests that 6784/2000/ARN also belongs to genotype P[7].

Although we did not sequence the minimum 500 bp/gene, we propose a tentative genotype classification based on ≈300–350 nucleotides sequenced by using the scheme of Matthijnssens et al. (*11*). VP1-, VP2-, VP3-, VP6-, NSP1-, NSP2-, NSP3-, NSP4-, and NSP5-encoding gene segments of strain 6784/2000/ARN form a close phylogenetic cluster with human and animal rotavirus strains of the Wa-like genogroup, respectively, in R1, C1, M1, I1, A1, N1, T1, E1, and H1 genotypes (*11*). Nucleotide sequences deposited in GenBank are FM179285 (VP1), FM179286 (VP2), FM179287 (VP3), FM179288 (VP4), FM179289 (VP6), FM179290 (NSP1), FM179291 (NSP2), FM179292 (NSP3), FM179293 (NSP4), FM179294 (NSP5), and EF218667 (VP7).

## Conclusions

Serotype G5 rotaviruses, which are common in pigs but also detected in horses and cattle, were identified in the 1990s in children from Brazil who had diarrhea (*12*). This serotype has also been reported in children with severe diarrhea in Paraguay, Cameroon, Argentina, Vietnam, and the People’s Republic of China (*2*,*13*,*14*), which suggests that G5, although uncommon overall in humans, is found worldwide. Partial molecular analyses showed that human G5 strains are reassortants with various genetic compositions. Some human G5 strains from Brazil, China (LL36755), and Vietnam (KH210) contain a genotype P[6] VP4 gene, but their other genes have not been characterized (*12*–*14*). The novel 6784/2000/ARN strain characterized here shares a VP6 subgroup II specificity and a long RNA electrophoretic pattern with prototype human G5 strain IAL-28 but differs in subgroup and electropherotype from the Cameroon isolate MRC3105 (*10*). Strain 6784/2000/ARN has a P[7] VP4 genotype and represents a human strain with this VP4 specificity.

Detection of G5 rotaviruses with different genetic compositions from children in Cameroon raises questions about the origin of these strains. MRC3105 not only represents a reassortant strain between porcine rotaviruses and HRVs but also may have obtained gene segments from isolates of human Wa and DS-1 genogroups, as suggested by unusual combinations in its RNA profile, subgroup specificity, and P type (*10*). In contrast, 6784/2000/ARN seems to have obtained its outer capsid combination from a porcine rotavirus, and its overall genomic composition showed genetic exchange between a porcine parental strain and a human strain of the Wa genogroup. We hypothesize that these 2 G5 isolates with identical VP7 genes in different HRV genetic backgrounds might be independent progenies of a porcine G5 rotavirus that was co-circulating with human DS-1–like and Wa-like strains at the time of identification of the G5 isolate in southwestern Cameroon. Additional sequencing of common porcine and human strains is required to elucidate mechanisms involved in generation of genetic diversity during reassortment of rotaviruses from 2 species.

Although G5P[7] strains might be common in pigs, strain 6784/2000/ARN is a novel representative of this antigen combination in humans. Similarities of some of its gene segments with those of porcine rotavirus strains suggest that ARN G5P[7] is an animal–human reassortant rotavirus in which a few genes are derived from human strains. Introduction of animal rotavirus genes into the genetic background of common HRVs has resulted in global spread of various genotype specificities, including G9 and G12. In these emerging human strains, DS-1 and Wa genogroups served as parental strains to carry the new antigenic variants on the background of old genotype specificities. Further, human G5 strains whose overall genomic composition is Wa-like have a wide geographic distribution and were considered clinically important HRVs in South America during the 1990s. Surveillance is needed to determine if G5P[7] strains on a Wa-like genetic background will spread to other African countries.

## Supplementary Material

 Appendix Figure Phylograms showing genetic relationships between known human and animal rotavirus strains from GenBank and partial nucleotide sequences of structural protein 1 (VP1), VP2, VP3, VP4, VP6, and VP7 genes and nonstructural protein 1 (NSP1), NSP2, NSP3, NSP4, and NSP5 genes of genotype G5P[7] rotavirus isolated from a child with diarrhea in Cameroon. Trees were drawn to scale and rooted with sequences of avian rotavirus strain PO-13. Significant bootstrap values (>80%) are indicated at branch nodes. Scale bars show 0.01 substitutions per nucleotide. Strain 6784/2000/ARN/Hu is in **boldface** in each phylogram. Hu, human; Po, porcine; Bo, bovine; Lp, lapine; Si, simian; Av, avian; Eq, equine; Ca, canine; Mu, murine; Ov, ovine; Rm, rhesus macaques; SG, subgroup.
