# Supplementary material for: Novel Human Rotavirus Genotype G5P[7] from Child with Diarrhea, Cameroon
Source: Emerg Infect Dis. 2009 Jan;15(1):83–6. doi: 10.3201/eid1501.080899 (PMC2662634; doi:10.3201/eid1501.080899)
Supplement: Appendix Figure — Phylograms showing genetic relationships between known human and animal rotavirus strains from GenBank and partial nucleotide sequences of structural protein 1 (VP1), VP2, VP3, VP4, VP6, and VP7 genes and nonstructural protein 1 (NSP1), NSP2, NSP3, NSP4, and NSP5 genes of genotype G5P[7] rotavirus isolated from a child with diarrhea in Cameroon. Trees were drawn to scale and rooted with sequences of avian rotavirus strain PO-13. Significant bootstrap values (>80%) are indicated at branch nodes. Scale bars show 0.01 substitutions per nucleotide. Strain 6784/2000/ARN/Hu is in boldface in each phylogram. Hu, human; Po, porcine; Bo, bovine; Lp, lapine; Si, simian; Av, avian; Eq, equine; Ca, canine; Mu, murine; Ov, ovine; Rm, rhesus macaques; SG, subgroup. [file 08-0899_appF-s1.pdf]

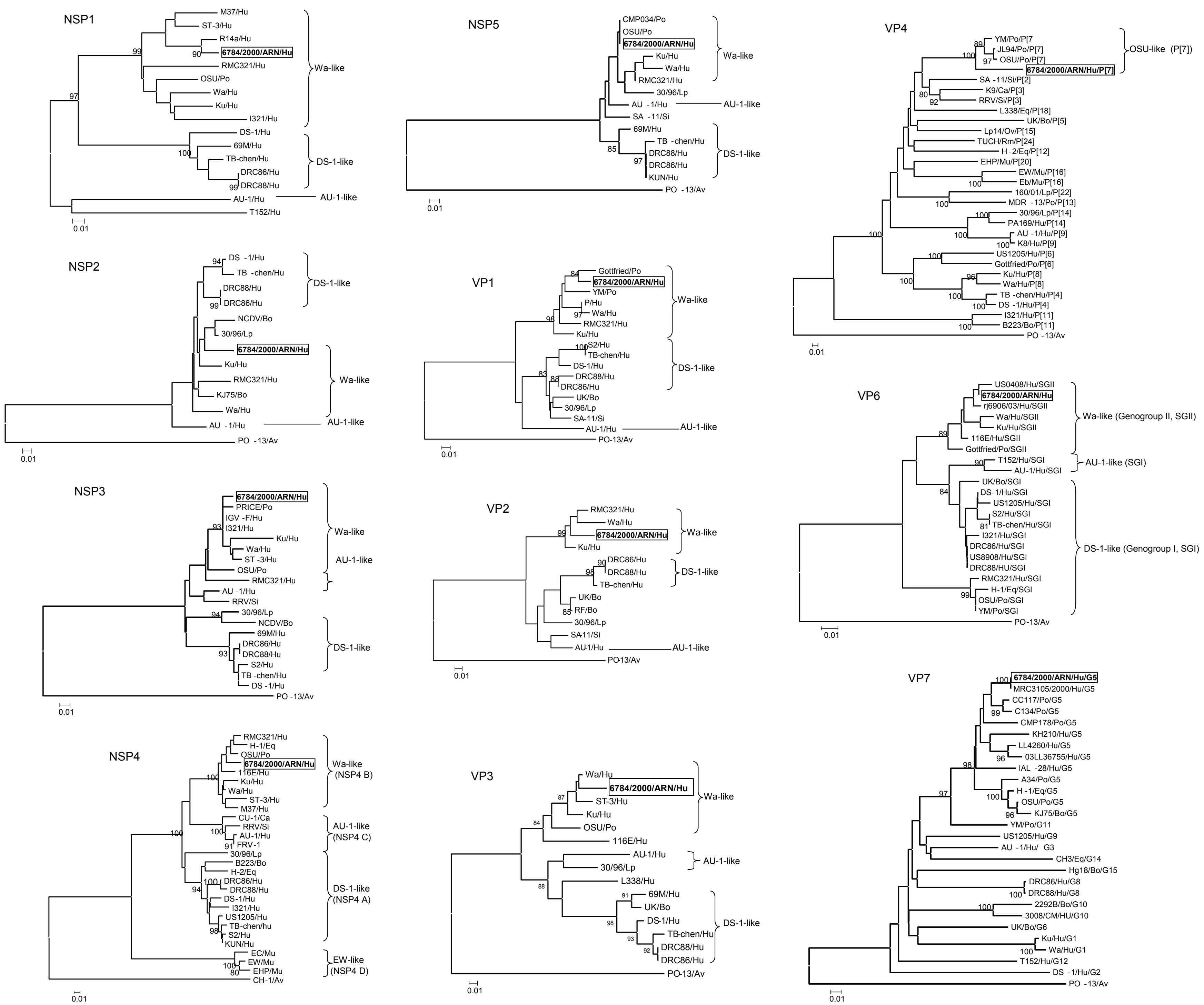
NSP1
M37/Hu
ST-3/Hu
R14a/Hu
6784/2000/ARN/Hu
RMC321/Hu
OSU/Po
Wa/Hu
Ku/Hu
I321/Hu
Wa-like
DS-1/Hu
69M/Hu
TB-chen/Hu
DRC86/Hu
DRC88/Hu
DS-1-like
AU-1/Hu
AU-1-like
T152/Hu
99
90
97
100
99
0.01
NSP2
DS -1/Hu
TB -chen/Hu
DRC88/Hu
DRC86/Hu
DS-1-like
NCDV/Bo
30/96/Lp
6784/2000/ARN/Hu
Ku/Hu
RMC321/Hu
KJ75/Bo
Wa/Hu
Wa-like
AU -1/Hu
AU-1-like
PO -13/Av
94
99
0.01
NSP3
6784/2000/ARN/Hu
PRICE/Po
IGV -F/Hu
I321/Hu
Ku/Hu
Wa/Hu
ST -3/Hu
OSU/Po
RMC321/Hu
Wa-like
AU-1-like
AU -1/Hu
RRV/Si
30/96/Lp
NCDV/Bo
69M/Hu
DRC86/Hu
DRC88/Hu
S2/Hu
TB -chen/Hu
DS -1/Hu
DS-1-like
PO -13/Av
93
94
93
0.01
NSP4
RMC321/Hu
H-1/Eq
OSU/Po
6784/2000/ARN/Hu
116E/Hu
Ku/Hu
Wa/Hu
ST-3/Hu
M37/Hu
Wa-like
(NSP4 B)
CU-1/Ca
RRV/Si
AU-1/Hu
FRV-1
AU-1-like
(NSP4 C)
30/96/Lp
B223/Bo
H-2/Eq
DRC86/Hu
DRC88/Hu
DS-1/Hu
I321/Hu
US1205/Hu
TB-chen/hu
S2/Hu
KUN/Hu
DS-1-like
(NSP4 A)
EC/Mu
EW/Mu
EHP/Mu
EW-like
(NSP4 D)
CH-1/Av
100
100
100
91
100
94
98
100
80
0.01
NSP5
CMP034/Po
OSU/Po
6784/2000/ARN/Hu
Ku/Hu
Wa/Hu
RMC321/Hu
30/96/Lp
Wa-like
AU -1/Hu
AU-1-like
SA -11/Si
69M/Hu
TB -chen/Hu
DRC88/Hu
DRC86/Hu
KUN/Hu
DS-1-like
PO -13/Av
85
97
0.01
VP1
Gottfried/Po
6784/2000/ARN/Hu
YM/Po
P/Hu
Wa/Hu
RMC321/Hu
Ku/Hu
Wa-like
S2/Hu
TB-chen/Hu
DS-1/Hu
DRC88/Hu
DRC86/Hu
UK/Bo
30/96/Lp
SA-11/Si
DS-1-like
AU-1/Hu
AU-1-like
PO-13/Av
84
97
98
100
83
88
0.01
VP2
RMC321/Hu
Wa/Hu
6784/2000/ARN/Hu
Ku/Hu
Wa-like
DRC86/Hu
DRC88/Hu
TB-chen/Hu
DS-1-like
UK/Bo
RF/Bo
30/96/Lp
SA11/Si
AU-1/Hu
AU-1-like
PO13/Av
99
90
98
85
0.01
VP3
Wa/Hu
6784/2000/ARN/Hu
ST-3/Hu
Ku/Hu
OSU/Po
116E/Hu
Wa-like
AU-1/Hu
30/96/Lp
AU-1-like
L338/Hu
69M/Hu
UK/Bo
DS-1/Hu
TB-chen/Hu
DRC88/Hu
DRC86/Hu
DS-1-like
PO-13/Av
87
84
88
91
98
93
92
0.01
VP4
YM/Po/P[7]
JL94/Po/P[7]
OSU/Po/P[7]
6784/2000/ARN/Hu/P[7]
OSU-like (P[7])
SA -11/Si/P[2]
K9/Ca/P[3]
RRV/Si/P[3]
L338/Eq/P[18]
UK/Bo/P[5]
Lp14/Ov/P[15]
TUCH/Rm/P[24]
H -2/Eq/P[12]
EHP/Mu/P[20]
EW/Mu/P[16]
Eb/Mu/P[16]
160/01/Lp/P[22]
MDR -13/Po/P[13]
30/96/Lp/P[14]
PA169/Hu/P[14]
AU -1/Hu/P[9]
K8/Hu/P[9]
US1205/Hu/P[6]
Gottfried/Po/P[6]
Ku/Hu/P[8]
Wa/Hu/P[8]
TB -chen/Hu/P[4]
DS -1/Hu/P[4]
I321/Hu/P[11]
B223/Bo/P[11]
PO -13/Av
89
100
97
80
92
100
100
100
100
100
100
100
100
96
100
100
100
0.01
VP6
US0408/Hu/SGII
6784/2000/ARN/Hu
rj6906/03/Hu/SGII
Wa/Hu/SGII
Ku/Hu/SGII
116E/Hu/SGII
Gottfried/Po/SGII
Wa-like (Genogroup II, SGII)
T152/Hu/SGI
AU-1/Hu/SGI
AU-1-like (SGI)
UK/Bo/SGI
DS-1/Hu/SGI
US1205/Hu/SGI
S2/Hu/SGI
TB-chen/Hu/SGI
I321/Hu/SGI
DRC86/Hu/SGI
US8908/Hu/SGI
DRC88/HU/SGI
RMC321/Hu/SGI
H-1/Eq/SGI
OSU/Po/SGI
YM/Po/SGI
DS-1-like (Genogroup I, SGI)
PO-13/Av
89
90
84
81
99
0.01
VP7
6784/2000/ARN/Hu/G5
MRC3105/2000/Hu/G5
CC117/Po/G5
C134/Po/G5
CMP178/Po/G5
KH210/Hu/G5
LL4260/Hu/G5
03LL36755/Hu/G5
IAL -28/Hu/G5
A34/Po/G5
H -1/Eq/G5
OSU/Po/G5
KJ75/Bo/G5
YM/Po/G11
US1205/Hu/G9
AU -1/Hu/ G3
CH3/Eq/G14
Hg18/Bo/G15
DRC86/Hu/G8
DRC88/Hu/G8
2292B/Bo/G10
3008/CM/HU/G10
UK/Bo/G6
Ku/Hu/G1
Wa/Hu/G1
T152/Hu/G12
DS -1/Hu/G2
PO -13/Av
100
99
96
98
97
100
96
100
100
100
0.01
